# Supplementary material for: Synthesis and Investigation of the Analgesic Potential of Enantiomerically Pure Schiff Bases: A Mechanistic Approach
Source: Molecules. 2022 Aug 15;27(16):5206. doi: 10.3390/molecules27165206 (PMC9416351; doi:10.3390/molecules27165206)
Supplement: Supplementary file 1 [file molecules-27-05206-s001.zip › molecules-1811257-supplementary.pdf]

molecules

Supplementary Materials

Synthesis and Investigation of the Analgesic Potential of Enantiomerically Pure Schiff Bases: A Mechanistic Approach

Hamid Hussain Afridi [1,2], Muhammad Shoaib [1], Fakhria A. Al-Joufi [3], Syed Wadood Ali Shah [1], Haya Hussain [2], Abid Ullah [2], Mohammad Zahoor [4,*] and Ehsan Ullah Mughal [5]

[1] Department of Pharmacy, University of Malakand Dir (Lower) at Chakdara, Chakdara 18800, Pakistan
[2] Department of Pharmacy, Shaheed Benazir Bhutto University Sheringal Dir (Upper), Dir 18000, Pakistan
[3] Department of Pharmacology, College of Pharmacy, Jouf University, Aljouf 72341, Saudi Arabia
[4] Department of Chemistry, University of Malakand Dir (Lower) at Chakdara, Chakdara 18800, Pakistan
[5] Department of Chemistry, University of Gujrat, Gujrat 50700, Pakistan
* Correspondence: zahoor@uom.edu.pk

S-Schiff Bases

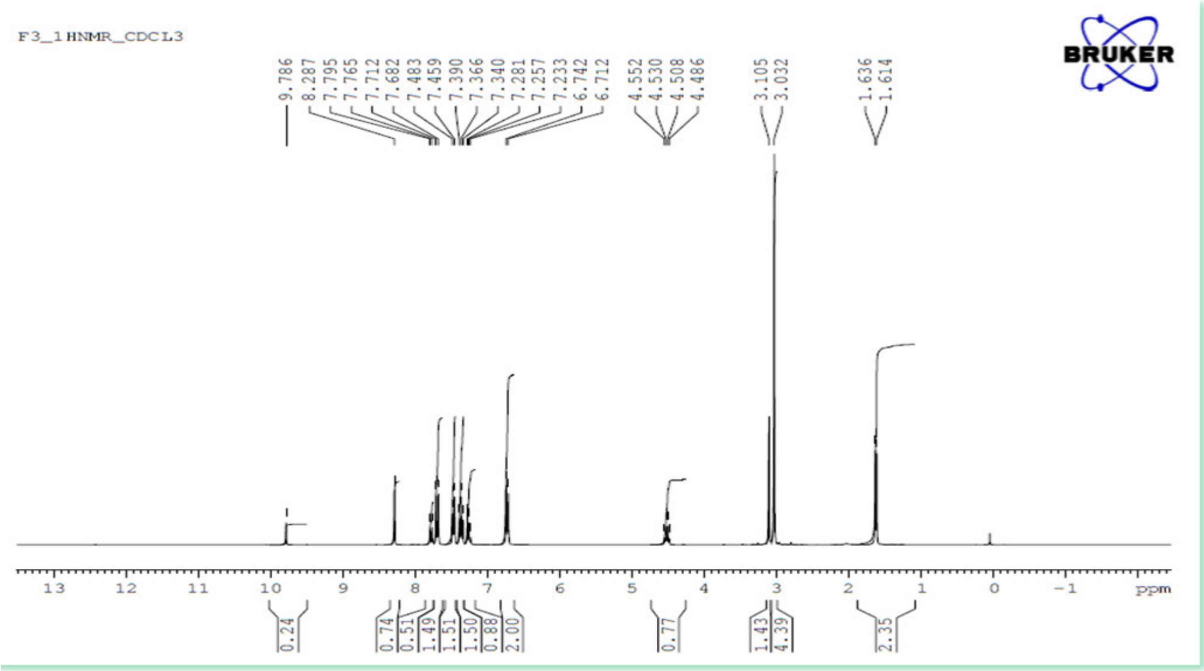

Figure S1. HNMR spectra of *(S,E)-N,N-dimethyl-4-(((1-phenylethyl)imino)methyl)aniline* **(H1)**

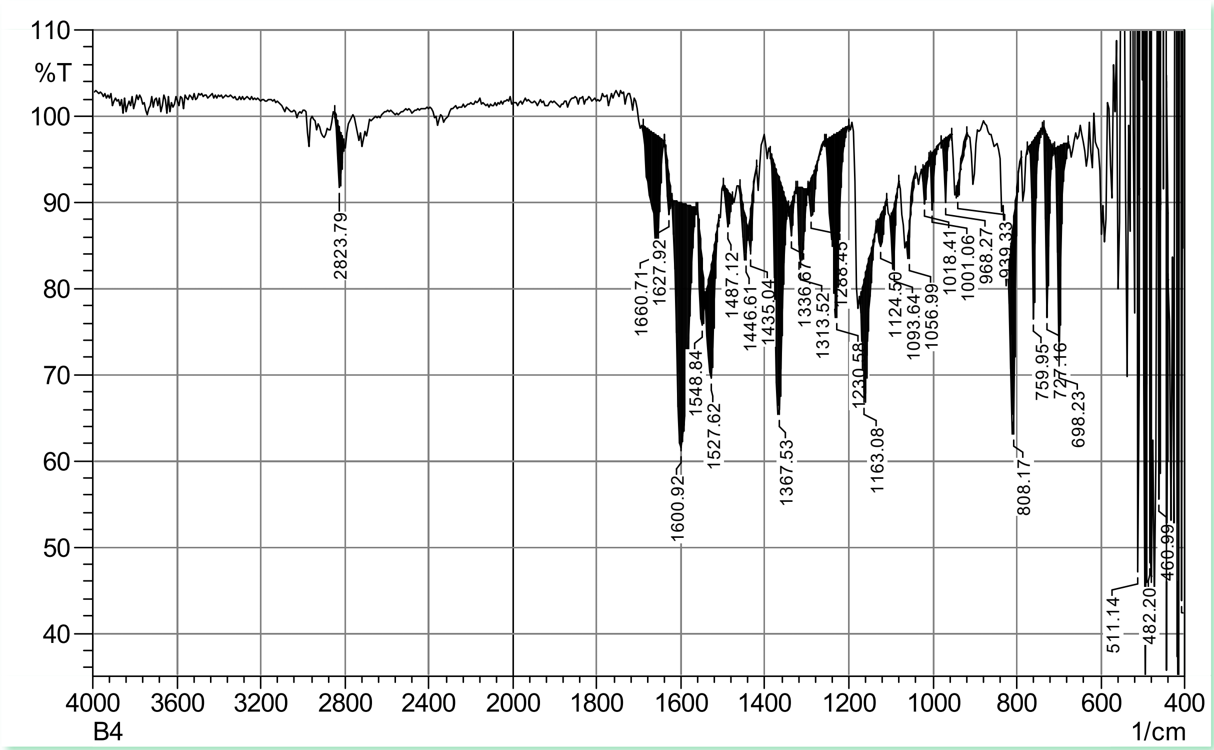

Figure S2. IR spectra of *(S,E)-N,N-dimethyl-4-(((1-phenylethyl)imino)methyl)aniline* **(H1)**

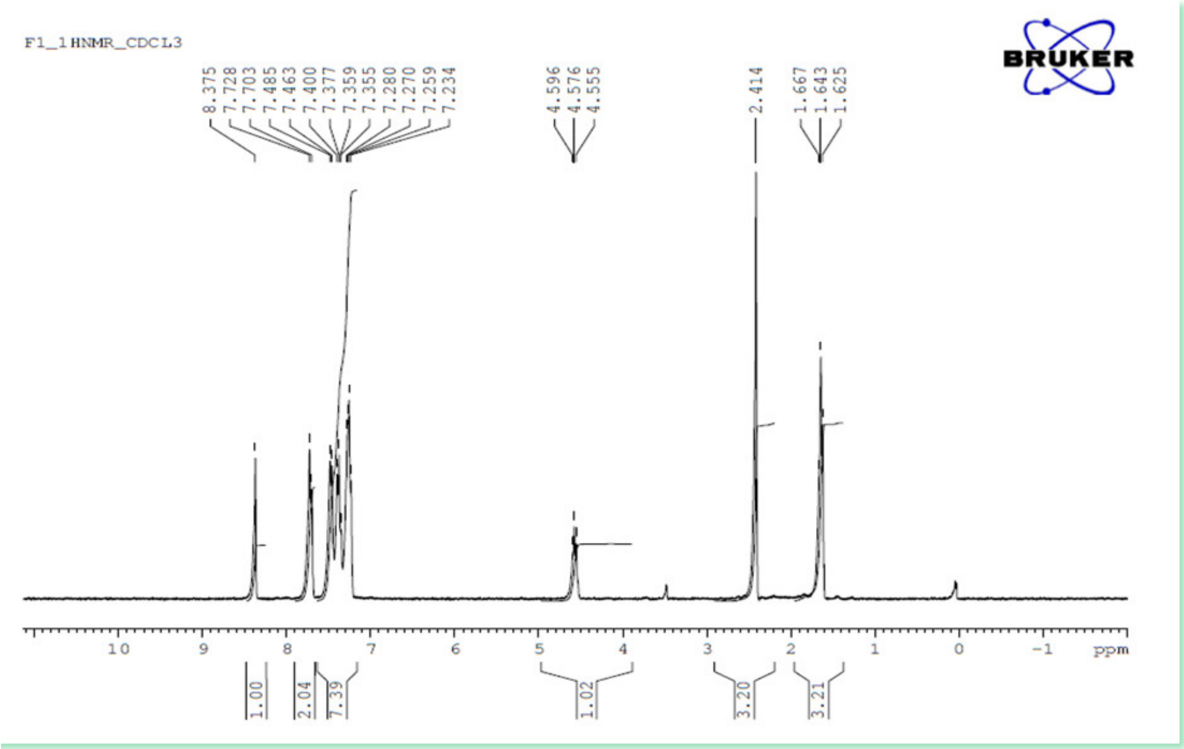

Figure S3. HNMR spectra of (S,E)-N-(4-methylbenzylidene)-1-phenylethanamine **(H2)**

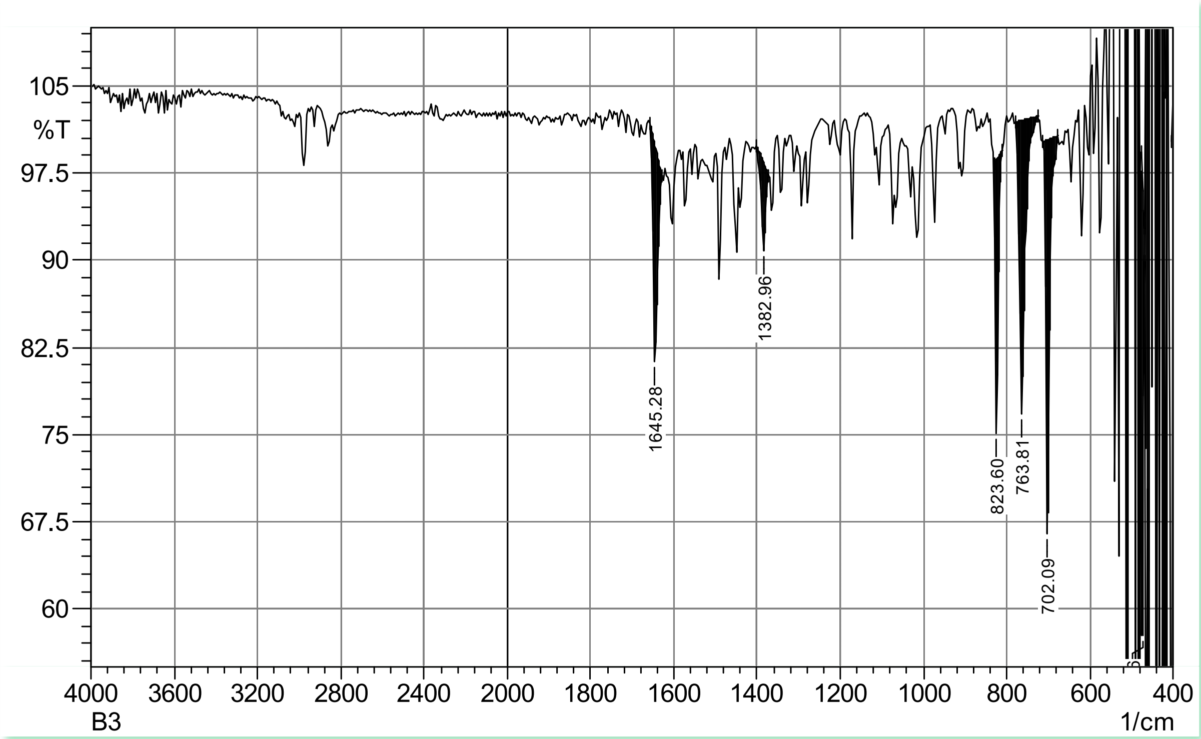

Figure S4. IR spectra of (S,E)-N-(4-methylbenzylidene)-1-phenylethanamine **(H2)**

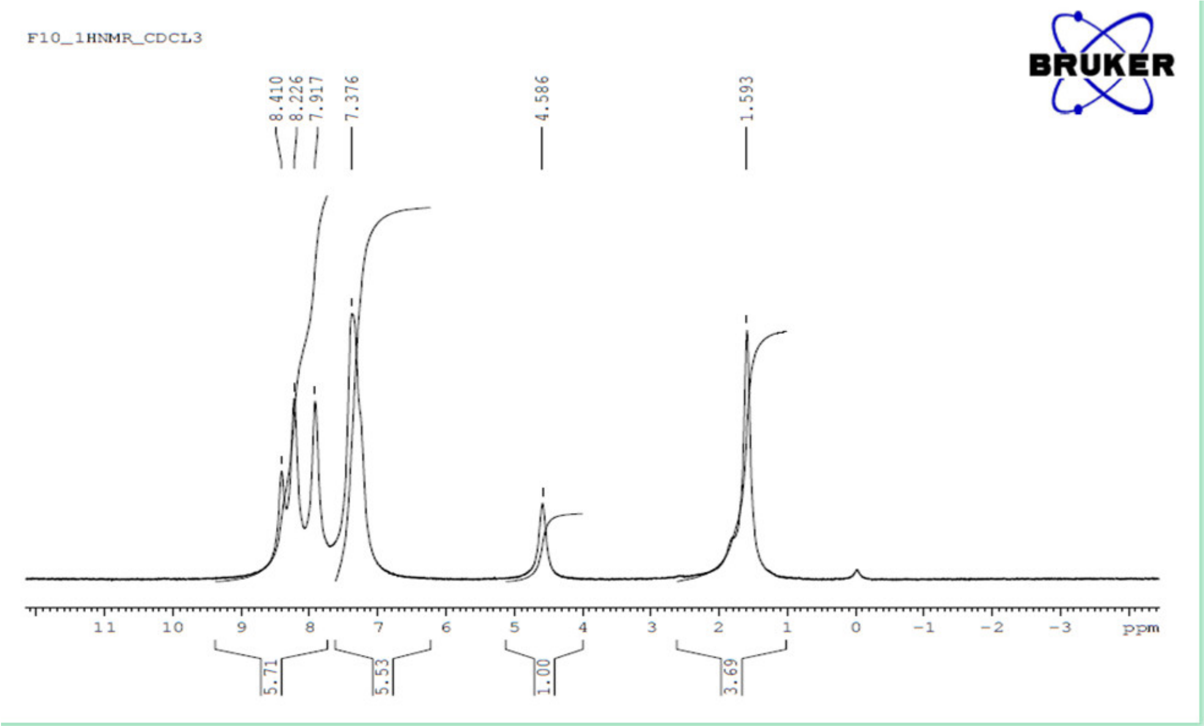

Figure S5. HNMR spectra of *(S,E)-N-(4-nitrobenzylidene)-1-phenylethanamine* **(H3)**

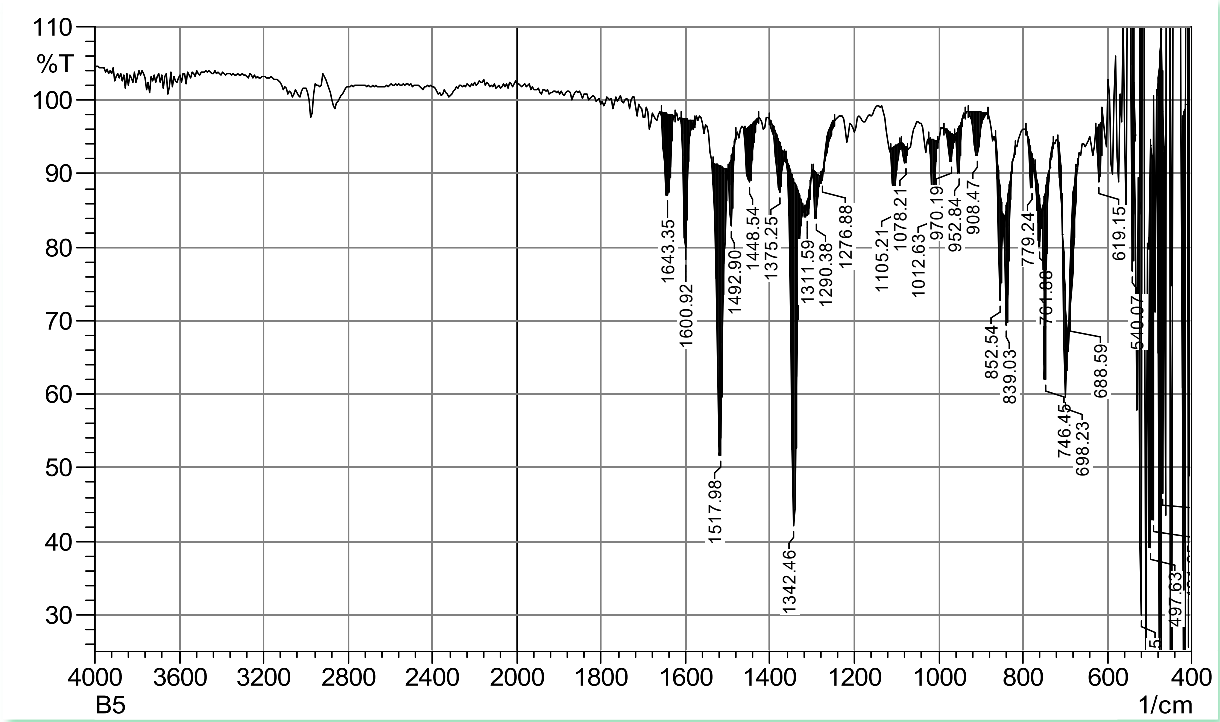

Figure S6. IR spectra of *(S,E)-N-(4-nitrobenzylidene)-1-phenylethanamine* **(H3)**

R- Schiff Bases

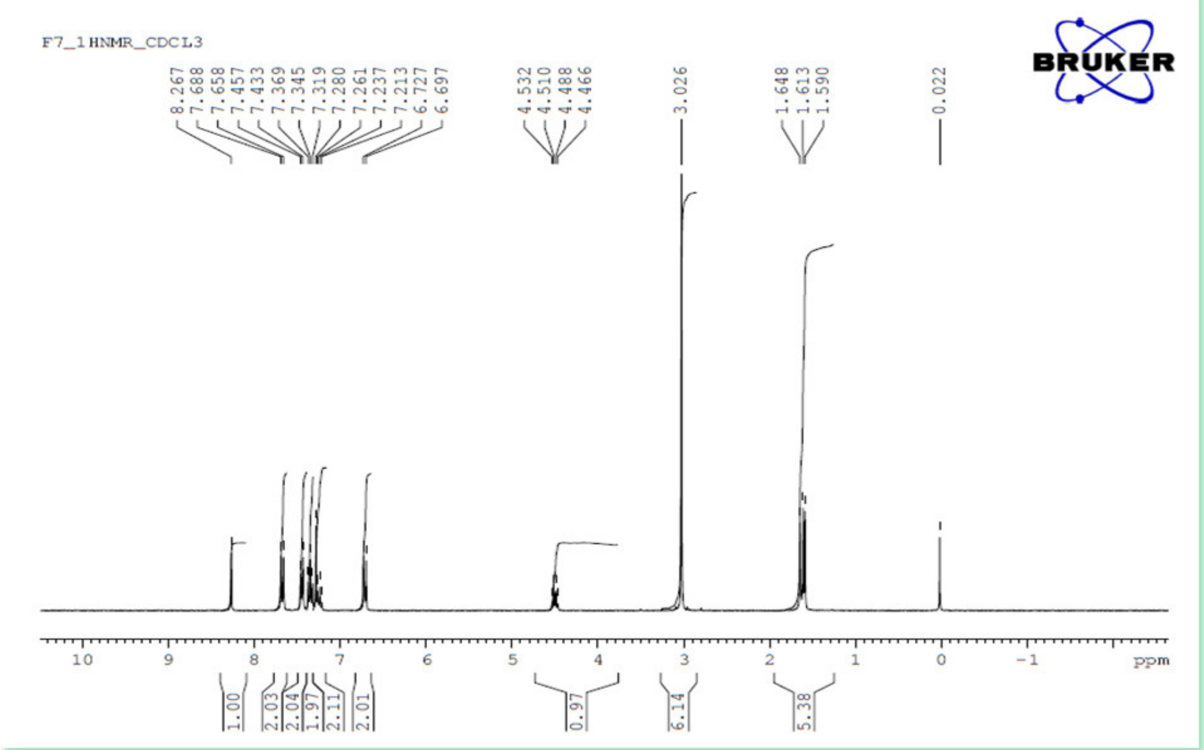

Figure S7. HNMR spectra of *(R,E)-N,N-dimethyl-4-(((1-phenylethyl)imino)methyl)aniline* **(H4)**

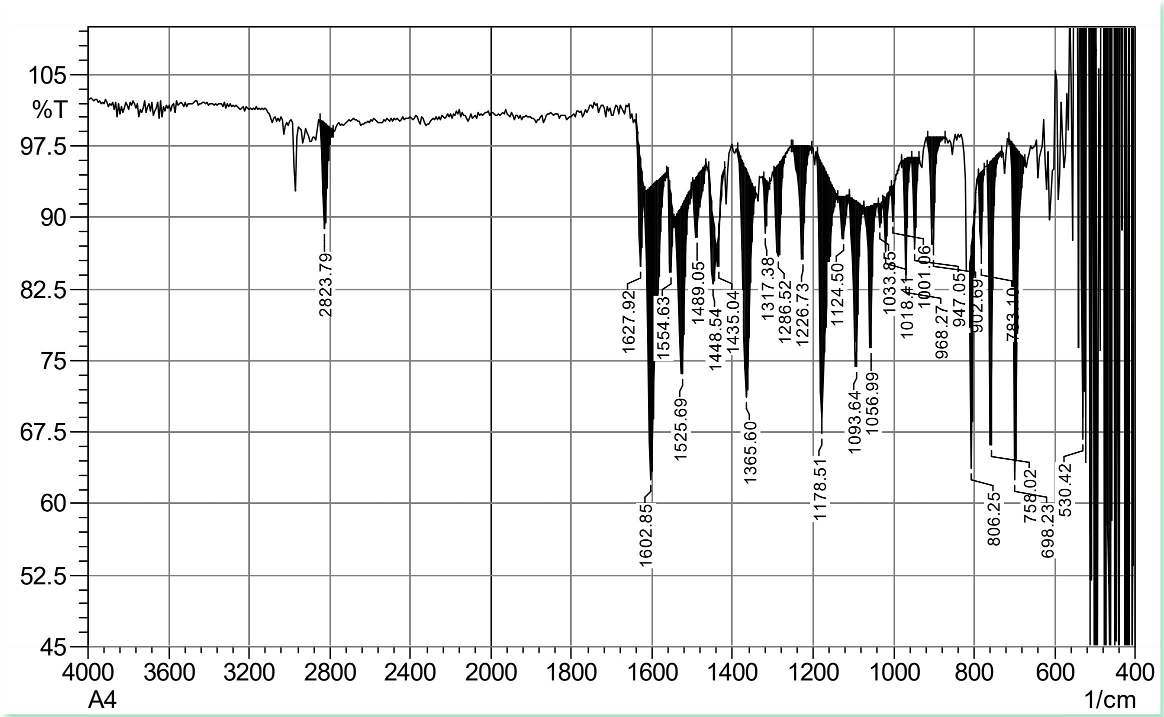

Figure S8. IR spectra of *(R,E)-N,N-dimethyl-4-(((1-phenylethyl)imino)methyl)aniline* **(H4)**

molecules

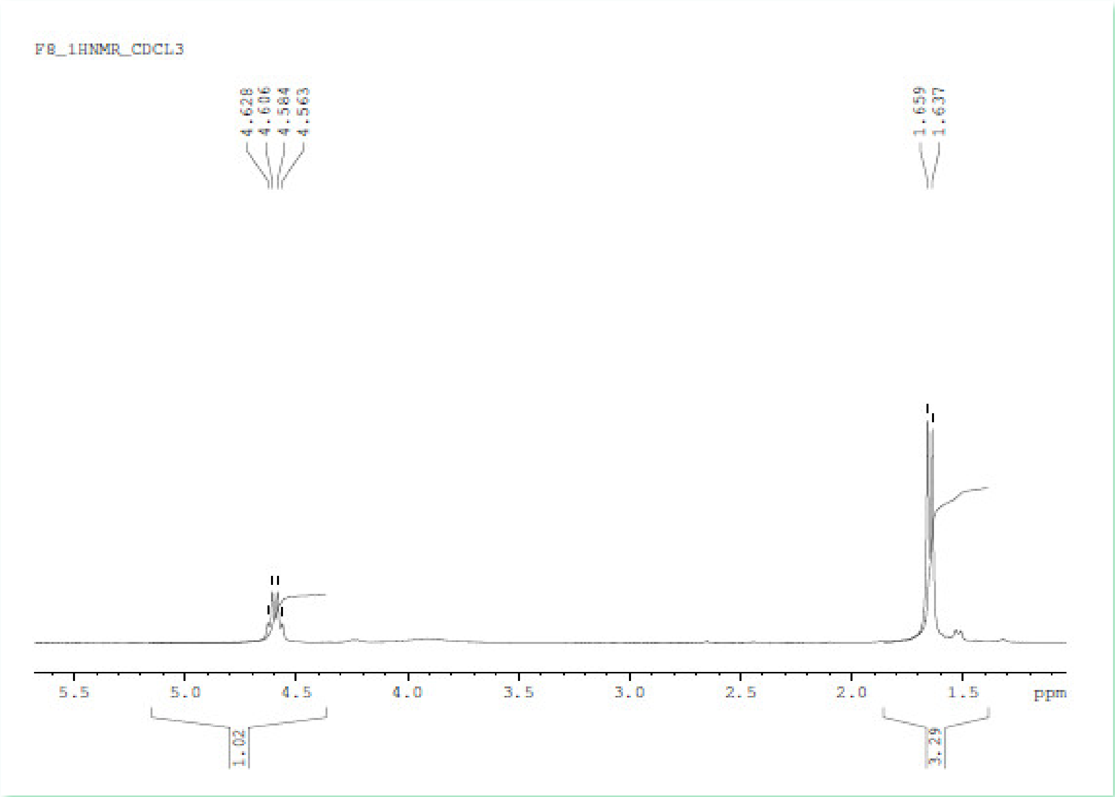

Figure S9. HNMR spectra of *(R,E)-N-(4-methylbenzylidene)-1-phenylethanamine* **(H5)**

molecules

MDPI

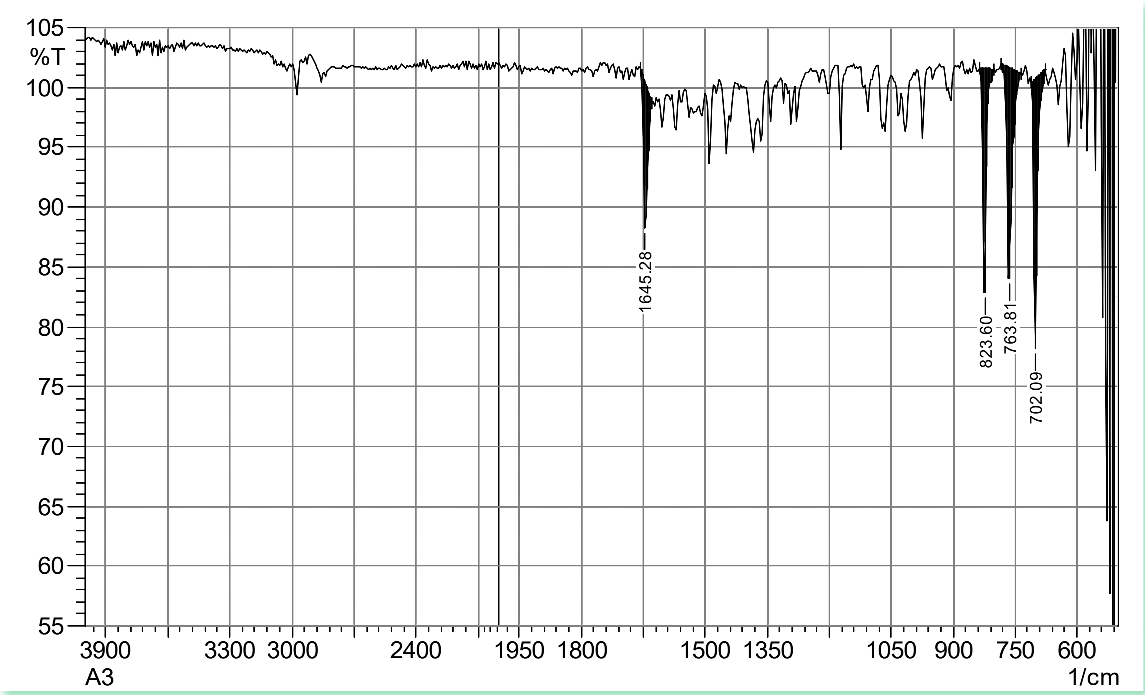

Figure S10. IR spectra of *(R,E)-N-(4-methylbenzylidene)-1-phenylethanamine* **(H5)**

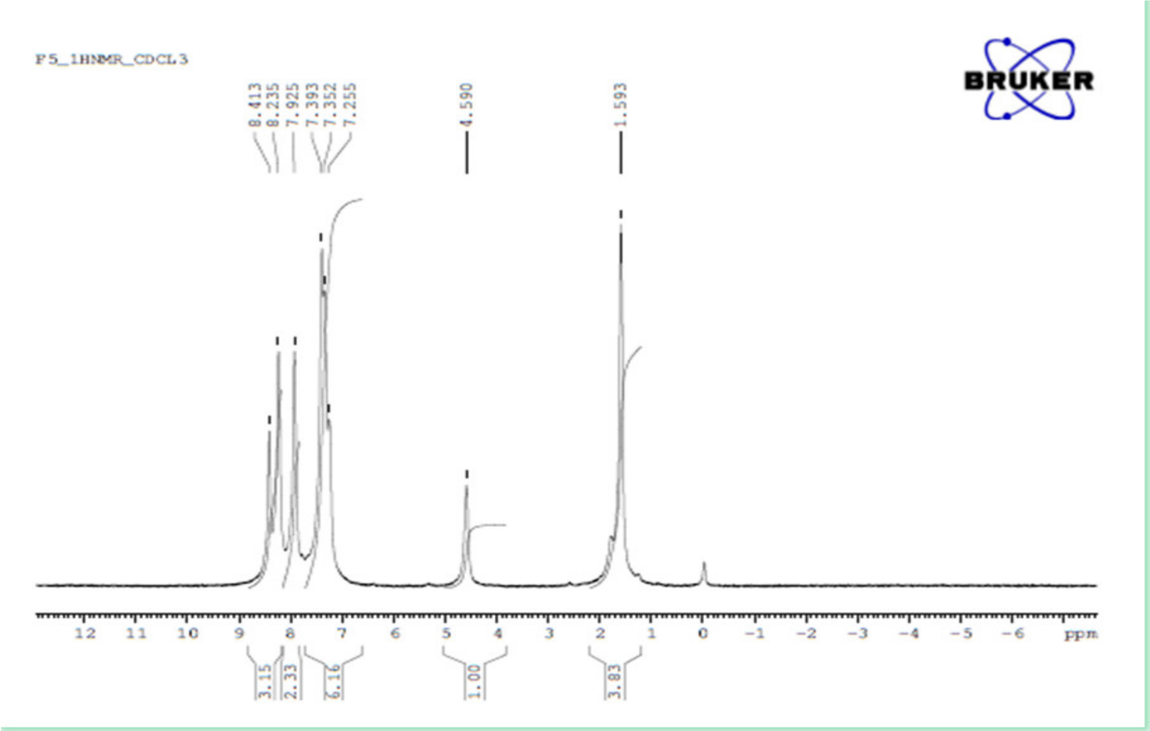

Figure S11. HNMR spectra of *(R,E)-N-(4-nitrobenzylidene)-1-phenylethanamine* **(H6)**

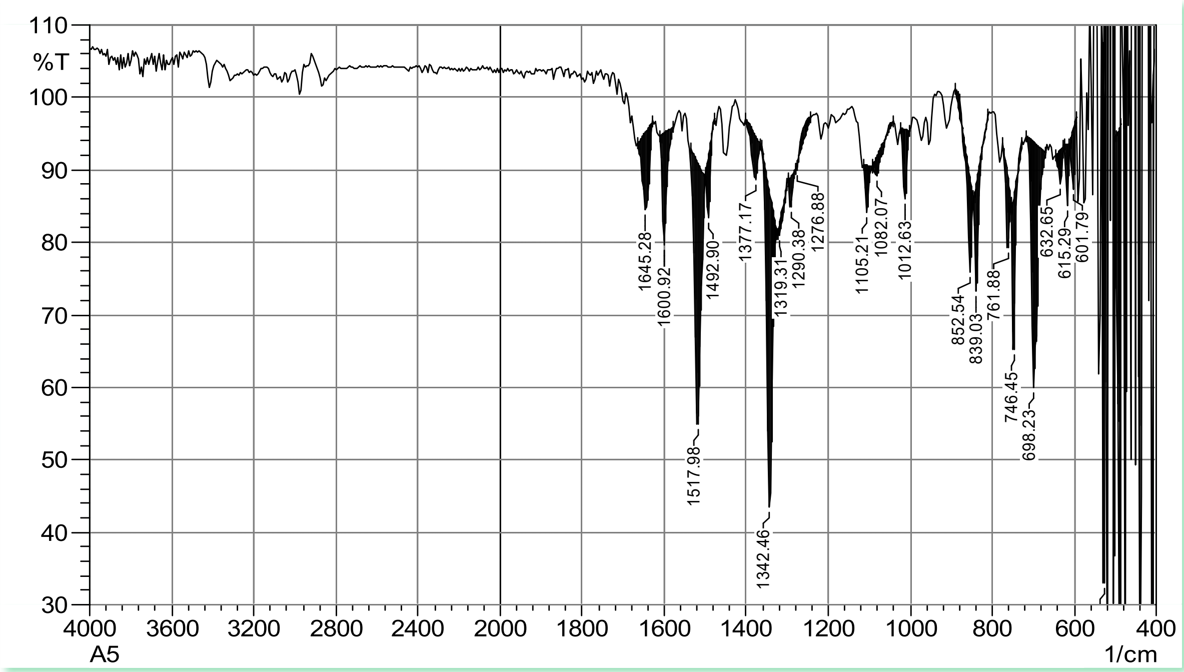

Figure S12. IR spectra of *(R,E)-N-(4-nitrobenzylidene)-1-phenylethanamine* **(H6)**
